# Videofluoroscopic swallow study training for radiologists-in-training: a survey of practice and training needs

**DOI:** 10.1186/s12909-022-03799-5

**Published:** 2022-11-07

**Authors:** Leah M. Coman, Elizabeth A. Cardell, John A. Richards, Amanda Mahon, Melissa D. Lawrie, Robert S. Ware, Kelly A. Weir

**Affiliations:** 1 Speech Pathology Service, Gold Coast Health, 1 Hospital Blvd, Southport, 4215 Queensland, Australia; 2 School of Health Sciences and Social Work, Griffith University, Queensland, Australia; 3 Menzies Health Institute Queensland, Griffith University, Queensland, Australia; 4 School of Medicine and Dentistry, Griffith University, Queensland, Australia; 5 Medical Imaging Department, Gold Coast Health, Queensland, Australia; 6 Allied Health Research, Gold Coast Health, Queensland, Australia

**Keywords:** Videofluoroscopic swallow study, Instrumental swallow assessment, Radiology, Registrars, Resident medical officers, Medical education and training, Dysphagia, Postgraduate

## Abstract

**Background:**

There is a lack of formal, published videofluoroscopic swallow study (VFSS) training targeting radiologists, yet radiology senior medical officers and resident medical officers (i.e., radiologists-in-training, known in Australia as “registrars”) are expected to be involved in VFSS interpretation of anatomical anomalies and reporting. This study investigated whether VFSS training is delivered to registrars during their specialist radiology training, whether it is a perceived need and, if so, to determine the desired content for inclusion in a targeted training package.

**Methods:**

A cross-sectional, mixed methods study design was used. An internet-based survey was circulated via convenience and snowball sampling to radiologists (both senior medical officers and registrars) and speech-language pathologists across Australia in October-November 2017. Surveys also were distributed to practitioners based in New Zealand and the United Kingdom, as they practised within similar health systems, and it was anticipated they may have similar VFSS training practices. The radiology survey contained 36 questions and the speech-language pathology survey contained 44 questions. Participants were asked the following: (1) Report their current VFSS radiology registrar training environment; (2) Advise whether radiology registrars need VFSS training; (3) Recommend the content, format, training intensity, and evaluation methods for an effective radiology registrar training package. Demographic data were analysed descriptively, and open-ended responses were analysed using qualitative content analysis.

**Results:**

21 radiology senior medical officers and registrars and 150 speech-language pathologists predominantly based at Australian tertiary hospital settings completed the survey. Most respondents (90.6%) identified that VFSS training is needed for radiology registrars. Only one speech-language pathologist respondent reported that they deliver VFSS training for radiology registrars. Specific content and teaching modalities for a VFSS training package, including diagnosing anatomical anomalies associated with dysphagia were recommended.

**Conclusion:**

While most of the radiologists and speech-language pathologists surveyed did not deliver VFSS training to radiology registrars, they identified that targeted training is needed to improve radiology registrars’ effectiveness and engagement in VFSS clinics. The training package content, format and evaluation methods recommended by participants will inform the development of a VFSS training package targeting radiology registrars to be piloted at an Australian tertiary hospital.

## Background

The videofluoroscopic swallow study (VFSS), also known as Modified Barium Swallow (MBS), is a dynamic fluoroscopic evaluation of swallowing where patients are imaged whilst ingesting a range of food and fluid consistencies to determine swallowing function and underlying pathophysiology [1]. The VFSS assesses movement patterns and coordination of swallowing-related structures of the upper aerodigestive tract and timing of swallow-respiratory events across the oral preparatory, oral, pharyngeal and upper oesophageal phases of swallowing [2]. Interpretation of VFSS involves making subjective visuo-perceptual assessment of images [3] and synthesizing results with knowledge of swallowing physiology and dysphagia to inform diagnosis, management recommendations and rehabilitation [4]. VFSS has been identified as a gold standard instrumental assessment of swallowing [5–11] and the most efficient and efficacious instrument for determining the management and rehabilitation of oropharyngeal dysphagia [12].

The VFSS clinic is interprofessional, usually consisting of a speech-language pathologist (SLP), radiographer, and radiologist. Models of care can differ across centres, depending on whether a radiologist is present for the VFSS procedure. Traditionally, in many countries, the VFSS clinic has been led by a radiologist and SLP [1, 13]. In Australia, some VFSS clinics are SLP-led in collaboration with a radiographer, with follow-up from a radiologist after the VFSS procedure [1]. Each profession contributes to the evaluation of swallowing. SLPs are experts in managing swallowing disorders. As such, SLPs critically analyse the functional aspects of the swallow, implement compensatory strategies, trial dysphagia rehabilitation techniques and may use VFSS images to deliver patient education. In traditional VFSS clinic models, the radiographer’s role can include image acquisition, fluoroscopy suite preparation, and monitoring radiation dosage [13, 14]. The radiologist provides medical diagnoses and identifies structural anomalies [1]. Speech Pathology Australia guidelines acknowledge that while not all work sites have access to a radiologist during the VFSS procedure, the combination of both radiology and SLP results in optimal diagnosis and adverse events arising during VFSS can be overseen by the radiologist [1]. Further, radiologists and SLPs in Australia have a legal requirement to document findings of the VFSS in the patient’s medical record [1].

SLPs undertake targeted competency training-to-criterion for interpreting and reporting VFSS images in assuring inter-rater reliability [18–23], and there is international variability around SLP VFSS training and heterogeneity across the methods, dosage and training environment [4]. Current VFSS training for SLPs include internet-based programs using case studies and quizzes to address the VFSS procedure, anatomy and physiology, swallow strategies and rehabilitation, interpretation, and reporting [18, 24]. For example, the MBS Measurement Tool for Swallow Impairment (MBSImP™), is a widely-used internet-based package for training quantified, standardized interpretation of swallowing impairment that has demonstrated inter- and intra-rater reliability post-training and external validity for SLPs [25].

Although there is a lack of universally accepted SLP competency indicators specific to VFSS, it is recommended that facilities adopt or develop their own formal competency guided by professional guidelines, local policies and published literature [1].

VFSS training for radiologists may not be as formal as SLP training, depending on VFSS training opportunities arising in residency programs [28]. Internet-based [30] and face-to-face [28, 31] training have been developed by SLPs for US radiologists and radiology residents and while they led to improved patient care [28, 31], inter-professional collaboration [31], and confidence in radiology resident VFSS interpretation [30], the details of the training and resources are not readily accessible outside of the US. “In-house” radiology registrar practical training has also been mentioned in UK literature without details of the training content [21]. Despite these outcomes, there remains a paucity of published, formalized VFSS training targeting radiologists.

In Queensland Health, Australia, registrars are resident medical officers who undertake an accredited course of study that leads to a higher medical qualification to become a senior medical officer (SMO) [33]. While local university medical programs (i.e., Bachelor of Medicine, Bachelor of Surgery – MBBS) promote an awareness of the contrast swallow procedure (upper gastrointestinal/standard barium swallow study) and extensive teaching for head and neck anatomy, there is little formal teaching and training around the diagnosis, assessment, and management of dysphagia. According to local programs, postgraduate radiology training, which is five years in duration, is vast in its scope. There are no dedicated lectures on swallowing disorders and assessment; rather, they are covered within multiple topics. In our local hospital settings, radiology registrars may be more accessible than SMOs in the hospital-based setting and therefore may be asked to comment on VFSS images during the VFSS procedure. In the absence of formal training, we decided to investigate radiology registrars’ confidence, knowledge, and level of training pre- and post-implementation of a dedicated VFSS training package, informed by a survey of practice to radiologists and SLPs.

The purpose of this study was to survey current practice and opinion of SMO radiologists, radiology registrars and SLPs across Australia, New Zealand, and the UK, given the paucity of evidence and detail around radiology VFSS training for trainee radiologists in the literature. Specifically, we aimed to:

1. Determine the status of current VFSS radiology registrar training.
2. Ascertain whether VFSS training is needed for radiology registrars.
3. Determine the content, format, training intensity, and evaluation methods for an effective radiology registrar training package.

It was hypothesised that current training methods would be ad hoc, with little use of formal structured training criteria, and that specific VFSS requirements would be considered essential to include in the training package.

## Materials and methods

### Study design and development

A cross-sectional, mixed methods study design was used. Two separate, purposive internet-based surveys for radiologists (SMOs and registrars) and SLPs were developed by the research team. The Checklist for Reporting Results of Internet E-Surveys (CHERRIES) [34] has been used to describe the development and implementation of the surveys. Each survey contained three sections, namely:

1. Demographic information about the respondents.
2. Information about existing radiology registrar VFSS training packages.
3. Recommendations around content for inclusion in a VFSS training package targeting radiology registrars.

Within the third section, respondents could select content and format for the training package via a list of options and provide additional information and/or suggestions via free-text comments. Mandatory questions such as “Does your centre have a dedicated VFSS training program for Radiology Registrars?” included an “unsure” non-response option to facilitate enforcement of mandatory questions and progression through the questionnaire. The survey questions were generated by members of the research team with expertise in VFSS, and the survey design was based on existing surveys of Australian clinical practice [35–38].

To ensure validity of the questions [39], each survey was reviewed and piloted. Initially, drafts of the surveys were circulated via email to local stakeholders external to the research team, comprising six SLPs with experience in VFSS and two SMO radiologists involved with registrar training at a tertiary hospital facility. Feedback was received from three SLPs for each survey and questions were updated accordingly. No feedback was received from the radiologists. Following the revisions, the surveys were circulated to the research team and same SLP and radiology external stakeholders via the SurveyMonkey® platform to pilot prior to wider circulation. Each survey was trialled twice by radiologists and SLPs, resulting in minor amendments following each trial. The radiologist survey contained 36 questions and the SLP survey contained 44 questions (the additional SLP questions pertained to demographical information). On average, each survey took seven minutes to complete. Demographic data included the experience and employment level of respondents, and employment levels were converted to a single system using the Queensland Health’s Health Practitioner Generic Level Statements so that they could be compared for statistical analysis. Adaptive questioning was employed in the survey, to reduce the number of unnecessary questions (e.g., if a respondent indicated that VFSS training is not needed for radiology registrars, the survey skipped questions pertaining to recommended training package content to the end of the survey where the respondent was invited to provide comments regarding their opinion).

### Eligibility

To be eligible for inclusion, respondents to the radiologist survey must have been SMO radiologists or registrars training in radiology who may/ may not have undertaken VFSS training. SLP participants must have had completed VFSS competency training and had experience with conducting VFSS clinics (ranging from less than one year to more than 16 years’ experience).

### Sampling method and sample size

Convenience sampling also employed, with SurveyMonkey® links circulated via email to radiology and SLP professional groups and networks known to the chief investigator and one of the primary investigators across Australia. Radiologists based at the first author’s work site (a tertiary hospital) were also provided with hard copies of the survey link to manually enter in their internet browser and were encouraged to circulate the link among their clinical networks. Surveys were also distributed via email through the New Zealand Speech-language Therapists’ Association, and two colleagues (one radiologist, one SLP) in the UK, due to similar health systems and VFSS practices to Australia. The snowball sampling recruitment method was employed following convenience sampling to maximise the number of respondents for each survey [37], as anyone with access to the SurveyMonkey® link could respond. It was anticipated that the total number of respondents would be between 70 and 110, based on similar research methodology for the SLP population [34–36] and radiologists [32]. Unique identifiers for each respondent were generated through the SurveyMonkey® platform to determine the number of survey participants. At the beginning of each survey, informed consent was obtained, and respondents were advised of the purpose of the study and data confidentiality and security as per ethical requirements.

### Confidentiality and data storage

A SurveyMonkey® subscription was purchased, with data accessible to the principal investigators only. All responses were confidential, except where respondents volunteered their name or provided sufficient details in the comments section that could lead them to be identified. At the completion of the survey, data was retrieved and saved in electronic format in a password-protected drive only accessible by the principal investigators and in a hard copy format in a locked filing cabinet in a locked storeroom in a speech pathology office at a tertiary hospital.

### Data collection period

The surveys were open for four weeks from the 9^th of^ October 2017 until 6th of November 2017. The radiologist survey was extended by three weeks until 27th of November 2017 to maximise the number of SMO radiologist and radiology registrar respondents, due to a low response rate.

### Statistical methods

#### Quantitative analysis

Data from each survey were imported from SurveyMonkey® into an excel spreadsheet and the IBM SPSS Version 24 program was used to perform statistical analysis. Demographic characteristics were summarised as frequency (%). The association between respondents identifying the need for VFSS training for radiology registrars and their employment grade or number of years competent in VFSS, were calculated using the Pearson chi-square test.

#### Qualitative analysis

Content analysis was performed on free-text comments in response to the open-ended questions:

Why do you think radiology registrars require VFSS training?

Why do you think that radiology registrars should NOT have VFSS training?

Any other feedback/comments that you would like to provide?

Content data analysis was undertaken as described by Graneheim and Lundman [40]. Free-text comments from each survey were exported from SurveyMonkey® into the NVivo Pro (Version 12) program, where responses were read and identified as separate meaning units. Meaning units were then coded, condensed (where necessary) and allocated into categories. Related subcategories were discussed and combined into overarching categories. From the categories, themes, the “essence” of the data [41], were developed. The data were initially analysed by the first author (with five years research experience) and verified by two additional investigators (with more than 20 years’ experience each), where consensus was reached.

## Results

Overall, 212 responses were received from 28 radiologists (SMOs and registrars) and 184 SLPs. Incomplete surveys (i.e., attempted by SLPs not trained in VFSS or Section 1 was incomplete) were deemed ineligible. Once these were removed, 21 radiologist and 150 SLP surveys remained. Of these, four radiology and 19 SLP surveys were incomplete, yet key components (i.e., all of Sect. 1 and “In your opinion, do Radiology Registrars require VFSS training?”) were completed and these surveys were included in the data analysis.

### Demographic data

Most respondents were based in Australia and worked in tertiary hospital settings (Table 1). There was a wide range of SLP clinical experience in VFSS (from less than one year to over 16 years) and employment level (from junior clinician level to director of SLP services). Over half (n = 87/150; 58%) of the SLP respondents had practiced for at least six years and most (n = 117/150; 78%) were senior SLPs. Both SMO radiologists (n = 8/21; 38.1%) and radiology registrars (n = 13/21; 61.9%) responded. Radiologists and SLPs reported that their centres predominantly provided VFSS services to adult-only populations (n = 99/171; 57.9%), then mixed populations (n = 61/171; 35.7%) and finally, paediatric-only populations (n = 11/171; 6.4%). Specific patient population age ranges reported by respondents are displayed in Table 2.

**Table 1:** Location of respondents, setting and type of centre

Location and Centre	Radiologists n = 21	SLPs n = 150
**Location**		
Queensland	18 (85.7%)	62 (41.3%)
New South Wales	2 (9.5%)	37 (24.7%)
Australian Capital Territory		4 (2.7%)
Victoria		17 (11.3%)
South Australia		11 (7.3%)
Northern Territory		1 (0.7%)
Tasmania		1 (0.7%)
Western Australia		8 (5.3%)
New Zealand UK	1 (4.8%)	4 (2.7%) 5 (3.3%)
**Type of Centre**		
Hospital – Quaternary	5 (23.8%)	18 (12%)
Hospital – Tertiary	16 (76.2%)	74 (49.3%)
Hospital – Secondary		25 (16.8%)
Hospital – Regional		26 (17.3%)
Hospital – Rural/Remote		3 (2%)
Private Practice		2 (1.3%)
Other		2 (1.3%)

**Table 2:** Populations receiving VFSS

Population	Radiologists n = 21	SLPs n = 150
Pre-term infants (< 37 weeks gestational age Infants (birth to 1 year) Toddlers (> 1 year to 3 years) Preschool (> 3 years to 4 years) School-aged (> 4 years to 17.99 years) Adults (≥ 18 years to 64 years) Adults > 64 years	0 (0%) 5 (23.8%) 6 (28.6%) 7 (33.3%) 7 (33.3%) 19 (90.5%) 17 (81%)	17 (11.3%) 56 (37.3%) 57 (38%) 54 (36%) 59 (39.3%) 140 (93.3%) 134 (89.3%)

A variety of VFSS clinic models were described, most commonly with a SLP led VFSS clinic with radiologists reviewing images outside of clinic (n = 89/171; 52%), and a joint SLP/radiology model (n = 64/171; 37.4%). Other models identified included: a radiologist and SLP model with no radiographer present; a SLP led clinic with radiologists only reviewing images and/or attending clinic on request; and a SLP led clinic with no radiologist involvement at all.

### Existing VFSS radiology registrar training

Most of the SLP respondents (n = 125/150; 83.3%) had access to formal SLP VFSS competency training programs at their facilities. This contrasted with the most of the reported VFSS training for radiology registrars, which was informal and ad hoc. Only one respondent, a SLP, reported implementing targeted VFSS training for radiologists at their site. The content delivered during the targeted training program depended on the level of experience of the radiology registrar and SLP present during the clinic. The training was delivered via several learning platforms, including face-to-face lectures, on-the-job training during VFSS clinic and small group tutorials through peer-based learning. Often, a senior radiology registrar was present during the VFSS training clinics; however, further details of radiology-delivered training were unknown. The respondent rated the training as 80% effective in their opinion and indicated that SLPs from their site would prefer to deliver a more formal training program.

### Is VFSS training needed for radiology registrars?

The opinion of most respondents (91.3% of SLPs, n = 137/150, and 85.7% of radiologists, n = 18/21) was that VFSS training is needed for radiology registrars. Pearson’s chi-squared test revealed no relationship between respondents identifying the need for training and their employment grade: radiologists (x²=2.154, p = 0.14); SLPs (x²=4.395, p = 0.62). Furthermore, there was no relationship between SLPs identifying the need for radiology registrar training and the SLPs years of VFSS competence/experience (x²=2.160, p = 0.83).

Many respondents who felt that there was a need for radiology registrar VFSS training illustrated this through free-text comments that were coded and allocated into the following categories:

1. Opportunities for and occurrence of VFSS training for radiology registrars.
2. Level of knowledge, skills, and confidence of radiology registrars in VFSS.
3. Enhancing interprofessional working and valuing the different professional roles in VFSS clinic.
4. Impact on patient care.
5. Potential outcomes of VFSS training.

**Fig. 1:**
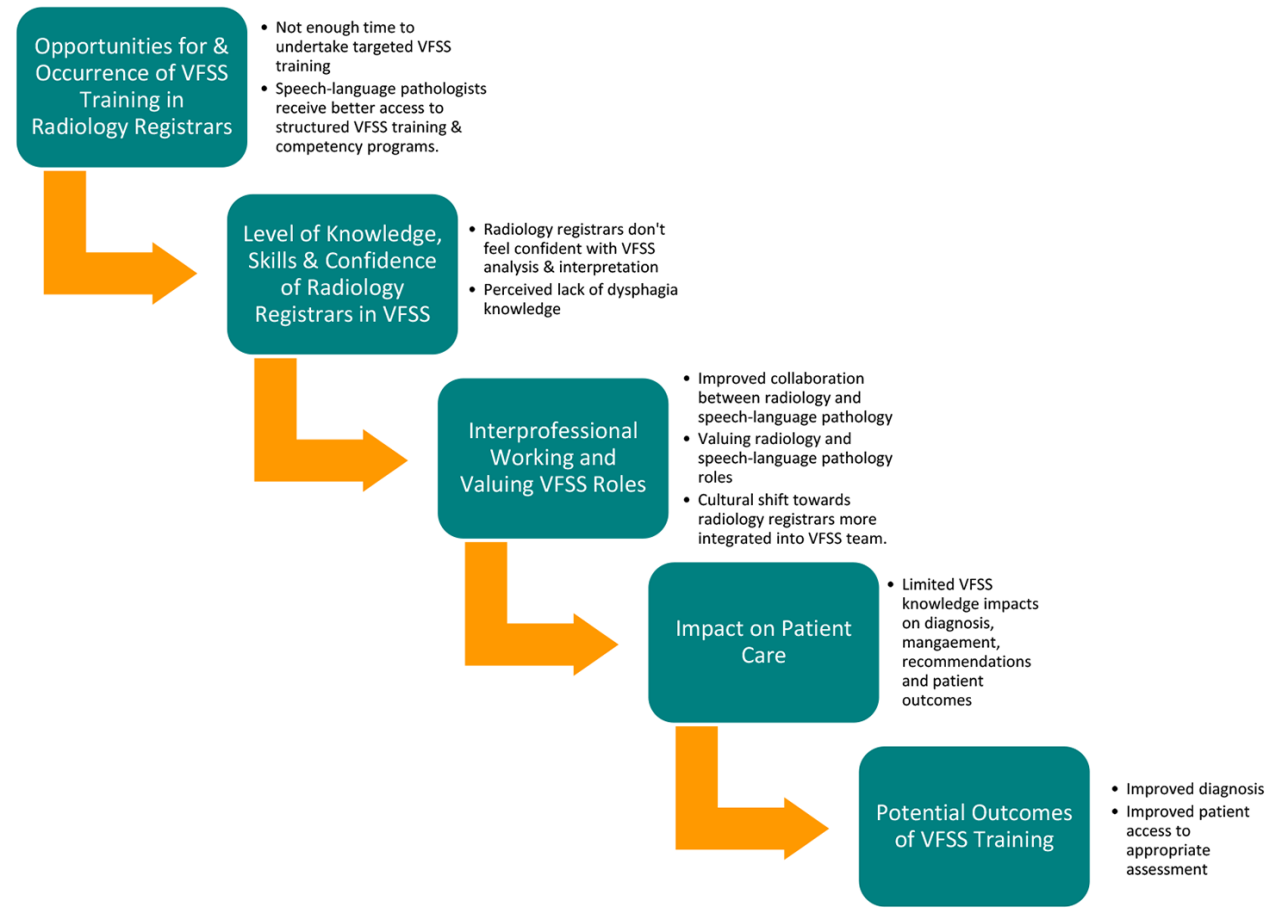
Flow diagram of categories—rationale for training

From these data, the themes of perceived current limited radiology registrar involvement in VFSS clinics and desired improvements (i.e., increased radiology registrar engagement and further understanding of VFSS purpose and enhanced clinical interpretation) to VFSS clinic were discovered. Comments supporting the need for training included “[VFSS] Should be more formal training such as with ultrasound, fluoroscopy and CT procedures.” (Radiology registrar, tertiary hospital, Queensland) and “It would be great to highlight the importance of their role and look at culture change in radiology registrars along with the clinical knowledge.” (SLP, regional hospital, New South Wales). Table 3 provides examples of coded and categorised comments. The categories that were developed from coded free-text responses align with a flow of processes that can be incorporated into a training methodology, which is represented by a schematic flow diagram (Fig. 1). This process flow demonstrates the impact that reduced training has on radiology registrars’ skill and confidence, the inter-professional roles within the VFSS clinic, risks to patient care and the potential outcomes that VFSS training would bring.

**Table 3:** Themes^1^, categories and samples of comments supporting VFSS training for radiology registrars

Categories	Number of Responses per Category	Respondents
**Opportunities for and occurrence of VFSS training for radiology registrars**	Radiologists: n = 2 SLPs: n = 9	**Perceived Current Limited Involvement of Radiology Registrars in VFSS Clinics** “In general, the training from the SPs^2^ is better than that provided by the radiologists.” (Senior medical officer radiologist, quaternary hospital, Qld^3^) “Should be more formal training such as with ultrasound, fluoroscopy and CT^4^ procedures.” (Radiology registrar, tertiary hospital, Qld) “The issue we have with our radiologist not wanting to review the VFSS is because he considers he is untrained and not competent at this. Thus, training radiology registrars would help solve this situation.” (Senior SLP, rural/remote hospital, NSW^5^)
**Level of knowledge, skills, and confidence of radiology registrars in VFSS**	Radiologists: n = 2 SLPs: n = 95	**Perceived Current Limited Involvement of Radiology Registrars in VFSS Clinics** “Currently most radiology registrars arrive thinking that they are just a foot on the pedal and the radiographer is probably just as good if not better than them.” (Senior medical officer radiologist, quaternary hospital, UK) Currently at our centre, we appreciate the registrars being present, however it is mainly a teaching experience for them versus actually providing useful information for us.” (Senior SLP, quaternary hospital, NSW) “Because they frequently do not wish to comment and have disclosed that they do not feel competent to report.” (Senior SLP, tertiary hospital, NSW)
**Interprofessional working and valuing VFSS roles**	Radiologists: n = 1 SLPs: n = 98	**Desired Improvements to Current VFSS Clinic** “Part of the challenge in a programme for radiology registrars is to show them how they add value with their broad medical knowledge. So, part of developing a training programme is to initiate a cultural shift, and a sense of clinical ownership.” (Senior medical officer radiologist, quaternary hospital, UK) “It would be great to highlight the importance of their role and look at culture change in radiology registrars along with the clinical knowledge.” (SLP, regional hospital, NSW) “To understand the purpose of a VFSS and their role in providing a radiologist opinion.” (Senior SLP, regional hospital, Qld)
**Impact on patient care**	Radiologists: n = 0 SLPs: n = 10	**Perceived Current Limited Involvement of Radiology Registrars in VFSS Clinics** “Whilst they can generally identify aspiration or pharyngeal residue etc., they have limited knowledge or understanding as to why this occurred. This can impact on information provided to patients, management plans and safety of oral intake for a patient.” (Senior SLP, regional hospital, Qld) **Desired Improvements to Current VFSS Clinic** “To ensure most accurate and appropriate analysis of VFSS with SP to ensure best patient care.” (SLP, quaternary hospital, NSW) “Objectively contribute to the interpretation of videofluoroscopic swallow findings and collaboratively ensure recommendations are made and agreed upon to enable improved clinical outcomes for patients.” (Senior SLP, tertiary hospital, WA^6^)
**Potential outcomes of VFSS training**	Radiologists: n = 2 SLPs: n = 29	**Desired Improvements to Current VFSS Clinic** “A number of education sessions from the SPs to both the registrars and consultants would be very valuable and would help also with interpretation of non VFSS barium swallows performed without a SP” (Senior medical officer radiologist, quaternary hospital, Qld) “Sometimes working in VFSS clinic, the most satisfying sessions are when the Rad reg^7^ is engaged and collaborative in their interaction with the SP. We value their role but at times a perception of disinterest and lack of importance may be inadvertently conveyed by them. This occurs infrequently, but if we could play a role in enhancing their skills in this area, perhaps this could improve the teamwork and outcomes for the pt^8^ in the session.” (Senior SLP, tertiary hospital, NSW) “Improves medical accountability for swallow dysfunction and the short and long-term consequences to respiratory health and nutrition.” (Senior SLP, tertiary hospital, WA)

In response to questions probing reasons why VFSS radiology registrar training is not needed, the themes of maximising existing resources and training, and maintaining professional roles and responsibilities emerged from the data. One radiologist stated, “It is like barium swallow, doesn’t need specific training just for VFSS.” (Radiology registrar, tertiary hospital, New South Wales). Further, 12 SLPs each provided justification against VFSS radiology registrar training, which were categorized as follows:

1. Additional cost and radiology registrar time.
2. Radiology registrar knowledge and (ad hoc) education already exist.
3. Over-stepping boundaries in VFSS clinic.
4. Radiology registrar training would not add value to VFSS clinic or patient care.

One SLP respondent acknowledged “They [radiology registrars] would benefit from knowing the rationale for choosing VFSS versus other instrumental swallow assessments however we fear that role boundaries may be blurred when it comes to actually conducting the VFSS procedure.” (Senior SLP, tertiary hospital, Victoria).

### Identified training requirements

**Fig. 2:**
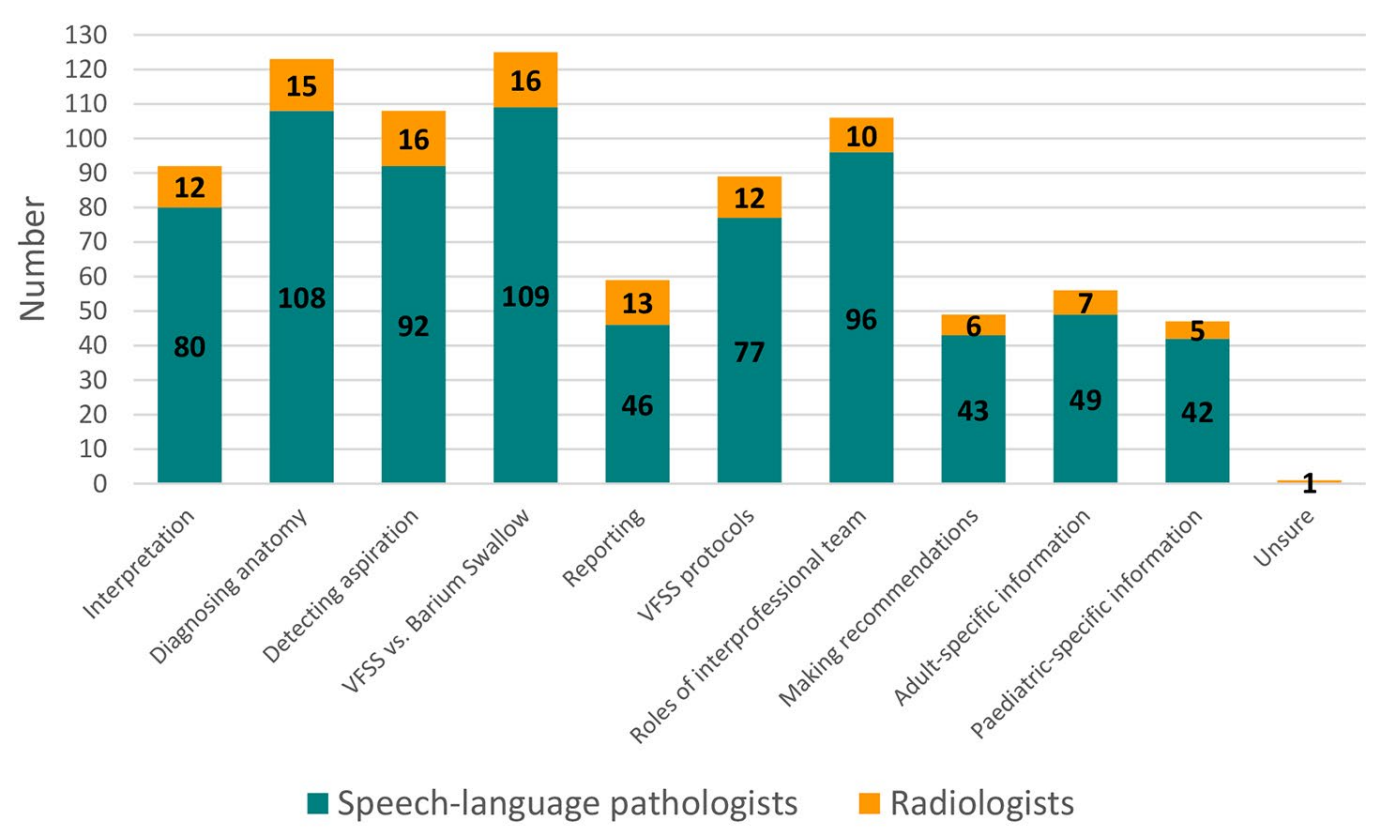
Content recommended for VFSS training package

Content identified, through frequency counts, as being important for a VFSS training package included:

1. Understanding the difference between VFSS and barium swallow/upper gastrointestinal studies.
2. Diagnosing structural or anatomical abnormalities impacting on swallowing and/or feeding function.
3. Detecting penetration of material into the laryngeal vestibule and aspiration of material into the trachea.
4. Understanding each of the VFSS clinic interprofessional team’s roles (Fig. 2).

A senior medical officer radiologist respondent suggested linking pathology to swallowing: “Understanding implication of common pathological conditions on swallow function – e.g., Zenker’s diverticulum, cervical osteophytes, strictures” (Senior radiology medical officer, quaternary hospital, Queensland). Approximately one third of SLPs (n = 42/150) recommended specific information for paediatric VFSS interpretation, for example: “Paediatric VFSS is different to adult. In relation to paediatrics - not always standard positions and changes during study, needing longer run time with some feeds or capturing beginning and middle of feed, ceasing film at the end of swallow not during, deep penetration versus aspiration, possible anatomical anomalies e.g., laryngeal cleft, TOF [tracheoesophageal fistula]” (Senior SLP, tertiary hospital, South Australia).

**Fig. 3:**
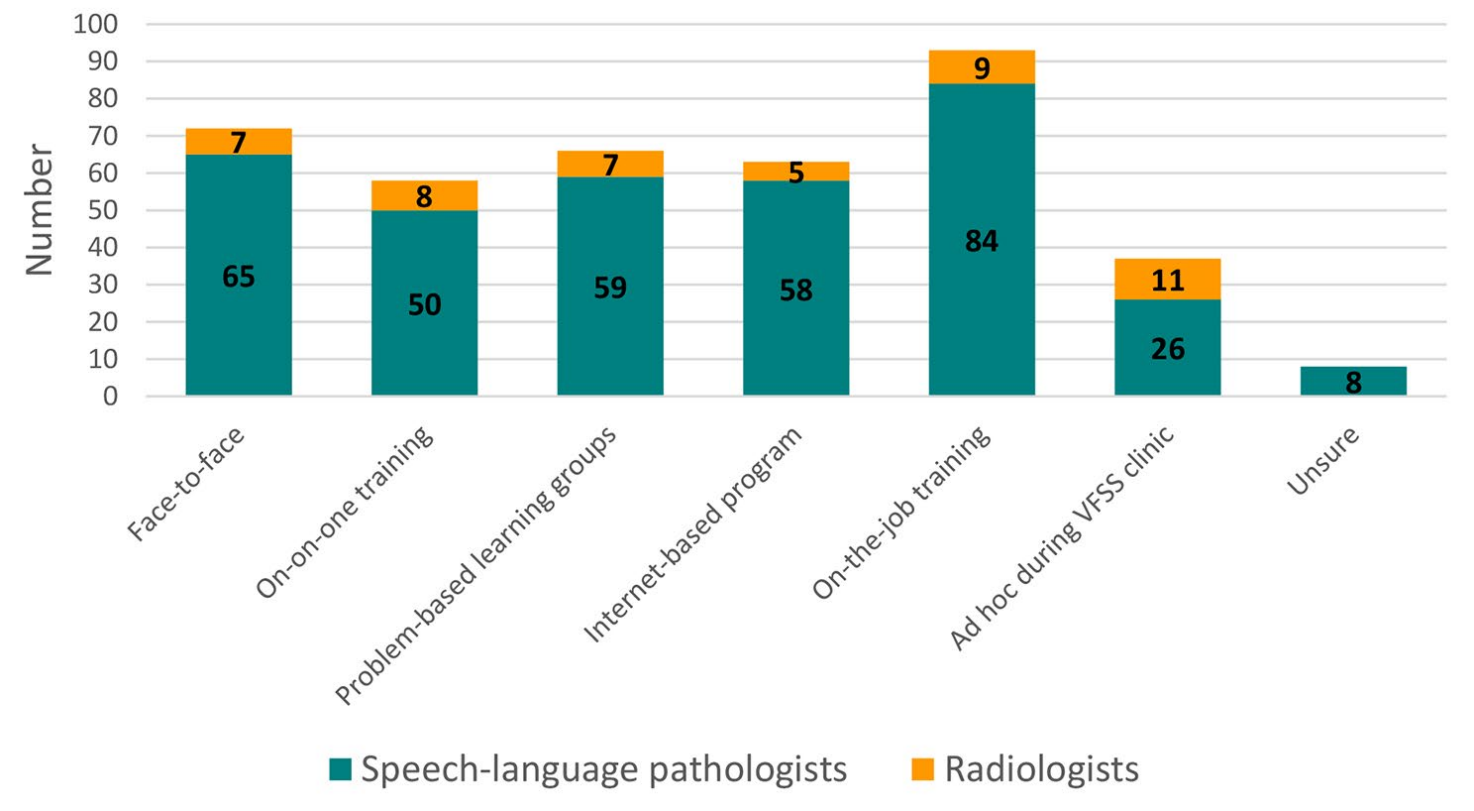
Training modalities recommended for VFSS training package

For specific training formats or modalities, both the SLP and radiologists’ preferences were distributed evenly across the options provided (Fig. 3). The most frequently identified modalities were:

1. On-the-job training during VFSS clinics.
2. Face-to-face didactic lectures provided to a group.
3. Problem (case)-based learning groups.
4. Internet-based or smart device application-based programs.

Respondents also recommended blended, combined training modalities: “I feel a series of didactic lectures would cover the necessary material, supplemented with involvement with several live cases to illustrate the didactic content” (Radiology registrar, tertiary hospital, Queensland) and “I certainly think however that training would need to encompass a minimum of theoretical learning either through face-to-face lectures, one-on-one training, problem-based learning groups and/or web-based programs, and also practical experience and competency-based assessment.” (Senior SLP, secondary hospital, Queensland).

### Duration of VFSS training and number of training sessions

The recommended duration of VFSS training in hours and weeks, and number of sessions are presented in Table 4. Overall, the recommendations for duration and sessions were similar for radiologists and SLPs: both respondent groups recommended a median of eight total number of training hours; radiologists recommended a mean of 5.05 weeks total duration of training and SLPs a mean total of 5.91 weeks; and a mean of six and five sessions were recommended by radiologists and SLPs, respectively.

**Table 4:** Duration and number of training sessions recommended for VFSS training

Professionals	Values	Total Number of Hours	Total Duration of Training (Number of Weeks)	Number of Sessions
**Radiologists**	mean ± SD median min-max	14.8 ± 19.95 8 2–80	5.05 ± 6.24 6 0.1–24	5.53 ± 5.10 6 1–20
**SLPs**	mean ± SD median min-max	12.43 ± 12.37 8 1–60	5.91 ± 5.97 4 0.1–30	8.41 ± 12.70 5 1-100

### Evaluation methods

To evaluate radiology registrars’ VFSS competency, the most frequently identified methods were:

1. Competency as determined by the supervising radiology senior medical officer.
2. Informal observation with case discussion.
3. Formal assessment.

**Fig. 4:**
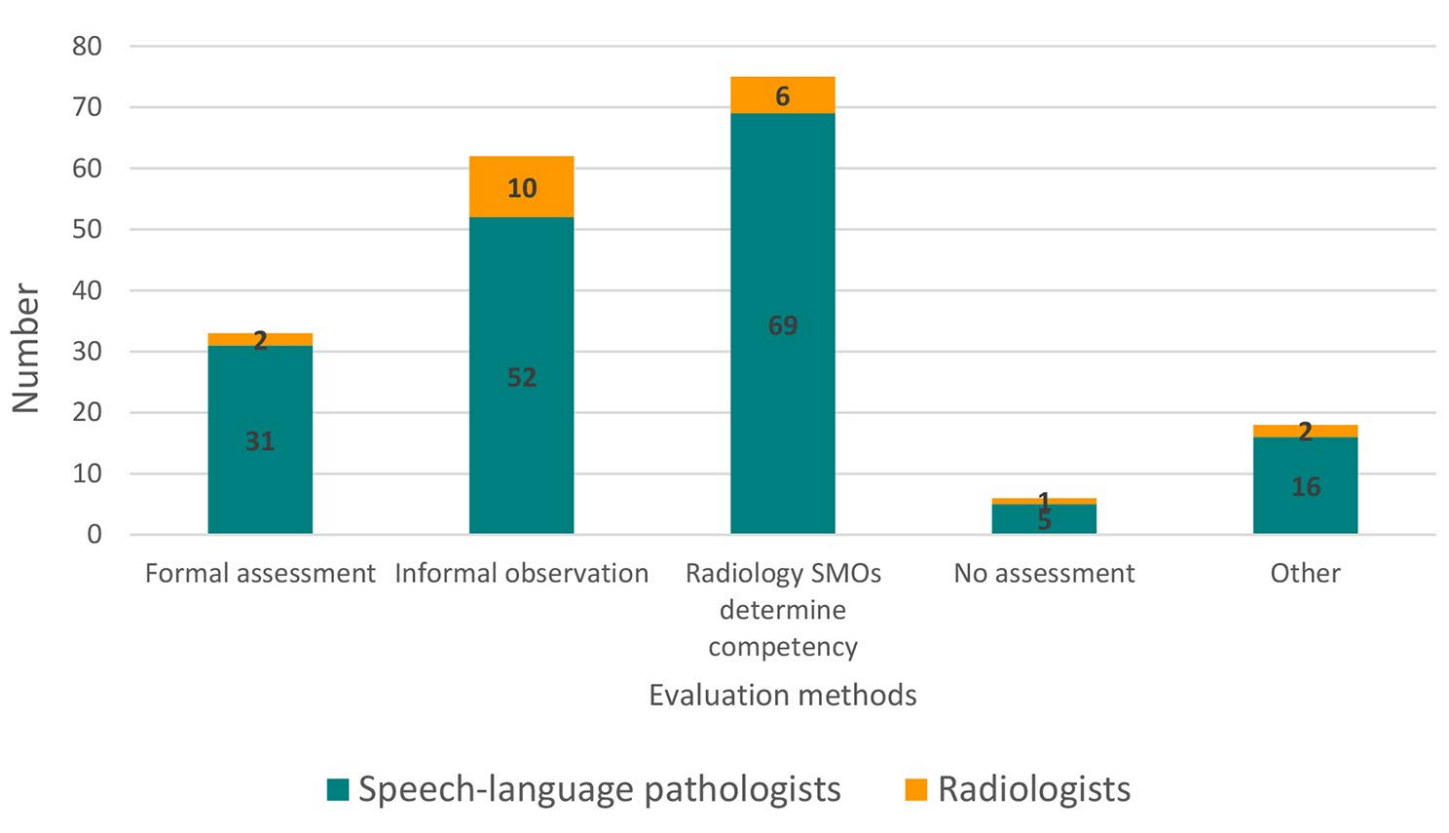
Evaluation methods recommended for VFSS training package

Other suggestions included collaborating with SLPs for assessment, online cases in assessment/exams, and multiple-choice quizzes, while only six respondents (SLPs n = 5; radiologists n = 1) indicated that no assessment process was needed (refer to Fig. 4).

## Discussion

A survey of SMO radiologists, radiology registrars and SLPs from across Australia was conducted to determine current practice and opinion regarding VFSS training for radiology registrars, and provide specific recommendations for content, format, and duration. Surveys were also disseminated to and received from participants working in New Zealand and the UK, as they practice in similar health systems. While most respondents agreed that VFSS training is needed for radiology registrars, all the radiologists and most of the SLPs reported there was no formal VFSS training available at their site, and only one SLP reported delivering training for radiology registrars. Therefore, our results support reports that VFSS training may not be formally and consistently undertaken with radiologists-in-training.

The theme of perceived current limited involvement of radiology registrars in VFSS clinics was prevalent in comments supporting the need for training. Reduced VFSS participation could be a factor in limiting radiology registrars’ willingness to engage in training, resulting in a perpetuating cycle (i.e., limited training leads to limited participation, leading to limited training opportunities). This also is represented by the process flow diagram generated from survey responses that reflects the impact that reduced training has on radiology registrars’ skill and confidence (refer to Fig. 1). Silbergleit and colleagues acknowledged that radiology residents may not have opportunities to engage in formal training VFSS at teaching institutions in the same manner as SLPs, while highlighting the importance of accuracy and reliability of image interpretation by all members of the VFSS team, due to the importance of safe oral intake. These authors recognised the need for formal agreement amongst radiologists, trainee radiologists and SLPs in VFSS interpretation. Through implementing a formal training lecture addressing normal and disordered swallowing using VFSS images and dysphagia terminology, improvements in radiology house officer VFSS interpretation were reported. A question that remains is why there is limited VFSS training available for radiology registrars across Australia, New Zealand, and the UK, when such training has been deemed important by most SLP and SMO radiologist and radiology registrar respondents in our study. One possible explanation is that VFSS clinic models differ across facilities. Although acknowledged that a combined radiologist and SLP team appears to optimize VFSS implementation, diagnoses, and management decisions, not all work sites have access to a radiologist to perform the procedure; therefore, SLP-led VFSS clinics are becoming an increasingly common model of practice. Whether the radiologist is present in the clinic or consulted following the clinic, the SLP’s scope of practice limitations in making medical diagnoses during VFSS must be considered as part of clinical governance.

The data collected for training content has provided useful information to inform the development of a VFSS training package for radiology registrars to redress the training gap that currently exists. A high proportion of radiology and SLP respondents recommended diagnosing anatomical abnormalities, understanding the difference between VFSS and barium swallow study, and detecting aspiration as key inclusions for the training package. Other content, such as making recommendations and specific information pertaining to adult and paediatric swallowing problems, was identified by respondents less frequently, as perhaps they are more related to SLP roles. Elements of successful SLP competency programs should also be incorporated into the radiology registrar training program, such as content targeting normal and abnormal swallowing, structural abnormalities contributing to dysphagia, and evaluating interpretation and reporting.

Technology and contemporary learning models feature in radiology training and the medical education literature. Internet-based radiology training resources using smart devices (e.g. iPads) to facilitate online self-directed learning are becoming increasingly popular with training residents in radiology and other areas of medicine, [42–46] One study discussed using internet-based teaching modules prior to face-to-face interactive sessions to ensure that basic knowledge is acquired, to optimise time spent with students with applied learning, and deliver cost effective teaching. Other studies have demonstrated that blended learning, that is, using a combination of online (e.g., internet-based/e-learning) and traditional face-to-face sessions, may be more beneficial for radiology and other healthcare professions compared to traditional didactic learning [47–49]. This aligns with contemporary models of educational design and delivery, such as flipped classroom learning where activities that have traditionally taken place inside the classroom take place outside the classroom, and vice versa (i.e. classroom activities and homework are interchanged) [50, 51]. This pedagogical approached to blended learning ensures that students become more active participants compared with students in traditional educational settings. The flipped classroom approach has been used in medical education and has demonstrated greater academic achievement than traditional lecture-based learning approaches [52]. The format recommendations for a VFSS training package aligned with the radiology training and medical education literature, [42–49, 53] in addition to known SLP VFSS training packages. Our findings suggest combined training modalities, such as face-to-face lectures and using internet-based or smart device application-based programs are preferable. In addition, on-the-job practical training through attending VFSS clinics and problem-based learning groups using real cases were also deemed important and need consideration as part of training package development.

The results of the survey have also provided a starting point for informed development of a training program for radiology registrars who do not have access to formal VFSS training, across Australia. The training program will contain content across adult and paediatric practice that is needed to increase radiology registrars’ understanding of professional roles, diagnostic accuracy, and interprofessional participation in the VFSS clinic. Further consultation to inform this development will be undertaken with local content experts, including senior medical officer radiologists involved with radiology registrar training, SLPs with expert knowledge in adult and paediatric VFSS interpretation, reporting and training, and university education design experts with experience in developing tertiary-level curriculum.

To our knowledge, this is the first study surveying radiologists and SLPs regarding current VFSS training for radiology registrars, the necessity for training, and identifying key components required for a training package targeting radiology registrars. However, there are several limitations with this study. Firstly, the survey was limited to Australia, New Zealand, and the UK, with most respondents based in Queensland, Australia. While identifying that training for radiology registrars was limited, respondents’ views on specifically why this was the case was not specifically raised. Such additional inquiry may have yielded further insights into the barriers/facilitators of radiology registrar training and participation in VFSS. Another limitation is the small radiology sample respondents, despite extended response time to maximise recruitment. As such, most information reflects SLPs’ responses, which do not present a balanced view across the professions regarding training requirements. Nonetheless, the information collected through this study has provided valuable insights into why VFSS training is needed for radiology registrars and specific details of what is required for training.

## Conclusion

In conclusion, this investigation has demonstrated that radiologists (SMOs and registrars) and SLPs agree that VFSS training is needed for radiology registrars. Development of a VFSS training package is underway and it is anticipated that completion of such a training package will improve radiology registrar diagnostic competence and confidence, facilitate co-ordinated and standardized interprofessional practice, and ultimately will lead to improved patient care.

## Data Availability

The datasets used and/or analysed during the current study are available from the corresponding author on reasonable request.

## Footnotes

1 Themes are highlighted in bold and italics within each category.

2 SLPs.

3 Queensland.

4 computed tomography.

5 New South Wales.

6 Western Australia.

7 Radiology registrar.

8 patient.

## Abbreviations

SLP: Speech-language pathologist
SMO: Senior medical officer
VFSS: Videofluoroscopic swallow study
